# Racial differences in serum chemokines in prostate cancer patients

**DOI:** 10.1002/cncr.35012

**Published:** 2023-09-12

**Authors:** Dev Karan, Jo Wick, Seema Dubey, Chandan Kumar-Sinha, Javed Siddiqui, Lakshmi P. Kunju, Kenneth A. Iczkowski, Arul M. Chinnaiyan

**Affiliations:** 1 Department of Pathology, MCW Cancer Center and Prostate Cancer Center of Excellence, Medical College of Wisconsin, 8701 Watertown Plank Road, Milwaukee, WI 53226, USA.; 2 Department of Biostatistics and Data Science, University of Kansas Medical Center, Kansas City, KS 66160, USA.; 3 Michigan Center for Translational Pathology, Department of Pathology, University of Michigan, Ann Arbor, Michigan 48109, USA.

**Keywords:** Prostate cancer, Racial disparity, Chemokines profile, CXCL2, CXCL5, CCL23

## Abstract

**Background:**

This study aimed to understand the differential levels of inflammatory chemokines in association with higher prostate cancer incidence and mortality in African American (AA) men than in Caucasians (CA).

**Methods:**

We utilized a chemokine assay to simultaneously measure 40 chemokines and cytokines levels in the serum of pre-operative prostate cancer patients and healthy controls of AA and CA races. Selected chemokines (CXCL2, CXCL5, and CCL23) serum level was validated in 211 serum samples from prostate cancer patients and healthy controls. Differential expression of CXCL5 and CCL23 was analyzed using immunohistochemistry in a representative cohort of prostate tumor tissues of AA and CA races.

**Results:**

Race-specific comparisons from 211 serum samples showed significantly higher levels of CXCL2 (control: 3104.0 pg/ml vs. cancer: 2451.0 pg/ml) and CXCL5 (control: 5189.0 pg/ml vs. cancer: 5459.0 pg/ml) in AA men than in CAs (CXCL2; control: 1155.0 pg/ml vs. cancer: 889.3 pg/ml, and CXCL5; control: 1183.0 pg/ml vs. cancer: 977.5 pg/ml). CCL23 differed significantly within and between the races with a lower level in AA cancer cases (454.5 vs. 966.6 pg/ml) than healthy controls (740.5 vs. 1263.0 pg/ml). Patient age, PSA, or Gleason scores were not significantly associated with these chemokines. Immunostaining for CXCL5 and CCL23 in a representative cohort of archival prostate tissues displayed significantly higher CXCL5 in prostate tumors than in adjacent benign tissues, while CCL23 was non-detectable in most of the analyzed tumor tissues.

**Conclusion:**

Lower levels of CCL23 in AA prostate cancer patient sera and tumor tissues and high CXCL2 and CXCL5 may contribute to aggressive prostate cancer, as often seen in AA men. The disproportionate levels of serum chemokines associated with race warrant further exploration to improve equitability in precision oncology to benefit prostate cancer patients.

## Precis

This study found that serum chemokines CXCL2, CXCL5, and CCL23 are the most distinguished chemokines differentially expressed in men of African American and Caucasian races. The impact of such chemokines contributing to the disparity in prostate cancer biology among races needs further investigation.

## Introduction

Cancer health disparities represent a significant health issue in the United States and globally. Prostate cancer is one such disease with a higher incidence and death rate in African American (AA) men than in Caucasians (CA). There has been great optimism and progress in prostate cancer treatment over the last decade, yet an overwhelming majority of prostate cancer patients relapse, with an estimated 34,500 deaths in 2022 in the US^1^. Prostate cancer patients of AA race have been noted to suffer a higher demographic burden of prostate cancer incidence and twice the burden of mortality compared to CA patients^2^. The known risk factors in prostate cancer mortality include age, genetics, diet and lifestyle, unequal access to health care, and other modifiable or non-modifiable factors. Recent studies suggested decreased mortality burden due to prostate cancer among Black and White men after adjusting non-biological factors^3–5^. Nevertheless, these observations do not fully resolve the higher incidence, mortality, and aggressive nature of prostate cancer among AA men. Even after adjusting for non-biological factors, prostate cancer mortality rates remain significantly higher in AA men than in CA^6,7^. A five-year analysis revealed a 73% higher incidence rate and more than two times higher deaths due to prostate cancer in AA than in CA men^2^. Therefore, various biological factors, including the high systemic levels of inflammatory exposure associated with differential allostatic load^8,9^, may account for aggressive prostate cancer by altering tumor-specific immunobiology in AA men.

Emerging evidence supports that the differences in systemic inflammation and immune responses may be biological drivers of cancer disparities^10–12^. Prostate tumors of AA race displayed an increased level of IFN-γ related gene signatures, enriched with proinflammatory cytokines, higher immune content scores, and upregulation of IFN-inducible transmembrane protein (IFITM3)^13,14^. We also showed a higher circulatory MIC-1 (macrophage inhibitory cytokine, also known as growth and differentiation factor 15 or GDF15) in prostate cancer patients of AA race than in CA and demonstrated inflammation-associated regulation of MIC-1^15^. Inflammatory susceptibilities are postulated as a therapeutic target for reducing prostate cancer in AA men^16^. Other studies also demonstrated racial differences in cytokine gene polymorphisms, circulating immune biomarkers by race and inflammation-induced prostate cancer in AA, and B-cell receptor signaling^17–20^.

To further underscore the role of systemic inflammation in prostate cancer among AA and CA men, we profiled the serum levels of 40 chemokines and cytokines from the pre-operative prostate cancer patients and age-matched healthy controls. Several inflammation-associated chemokines and cytokines were noted differentially expressed in AA versus CA men. Serum levels of CXCL2 and CXCL5 exhibited race-specific differences, while CCL23 showed both race- and cancer-specific differences supporting the potential contribution of systemic chemokines in differential outcomes among races in association with prostate cancer.

## 2. Patients and Methods

### 2.1. Patients and Serum Samples:

211 serum samples from prostate cancer patients and healthy controls were tested. 120 of the serum samples (80 from prostate cancer patients and 40 healthy donors) were acquired from the University of Kansas Cancer Center as described previously^15^. Additional serum samples from 50 CA and 41 AA prostate cancer patients collected at the time of diagnosis were obtained from the University of Michigan following approval from the Institutional Review Board and Ethics Committee. The method of determining the subject’s race and health status is self-reported during clinical visits. The self-declared healthy donors (controls) are referred to as men free from any detectable disease, including cancer, and were not on any medications at the time of blood collection.

### 2.2. Cytokine-Chemokine Screening:

We used a 40-plex human chemokine assay kit (Bio-Rad Hercules, CA, USA) to determine the differential level of cytokines-chemokines among CA and AA men associated with prostate cancer. This assay measured 40 different chemokines and cytokines (CCL1, CCL2, CCL3, CCL7, CCL8, CCL11, CCL13, CCL15, CCL17, CCL19, CCL20, CCL21, CCL22, CCL23, CCL24, CCL25, CCL26, CCL27, CXCL1, CXCL2, CXCL5, CXCL6, CXCL8, CXCL9, CXCL10, CXCL11, CXCL12, CXCL13, CXCL16, CX3CL1, GM-CSF, IFN-γ, IL-1β, IL-2, IL-4, IL-6, IL-10, IL-16, MIF, and TNF-α) in a single assay. Briefly, the 40 analyte beads in an assay buffer were added to pre-wet wells. After the assay buffer wash, 50 μl of serum sample or standard was added to each well, and the experiment was performed per the manufacturer’s instructions. Data acquisition and analysis were completed using Luminex System (Austin, TX) and Bio-Plex Manager software (Bio-Rad).

### 2.3. Enzyme-Linked Immunosorbent Assay (ELISA):

Serum level of the selected chemokines CXCL2 (Catalog #27142, IBL, Japan), CXCL5, (Catalog #DX000, R&D Systems, MN), and CCL23 (Catalog #EHCCL23; Thermo Scientific) was validated using commercially available ELISA assay kits as per manufacturer’s instructions.

### 2.4. Immunohistochemistry (IHC) and Evaluation of Immunohistochemical Staining:

Tissue microarray (TMA) slides spotted in triplicates for the benign and cancer areas of prostate tumor tissues from AA and CA men were made with 3DHistech TMA grandmaster from prostatectomy blocks selected by the pathologist. The TMA included 15 and 16 evaluable matched cases of benign prostatic tissue for AA and CA prostate cancer patients. Immunohistochemistry was performed using an Omnis Autostainer (Agilent/DAKO, Santa Clara, CA). For detection of CXCL5, we used rabbit polyclonal antibody (Thermo Fisher: Product #710010), dilution 1:25, and HpH antigen retrieval overnight (16-hour incubation). To detect CCL23, we analyzed 20 prostate cancer tissues, ten each from AA and CA, using goat polyclonal antibody (sc-46070; Santa Cruz), dilution 1:150 (1 hour). CXCL5 and CCL23 staining intensity was recorded in a blinded fashion and graded on a four-point scale, as in our previous study^15^. The intensity scores on the TMA spotted in triplicates or quadruplicates were averaged.

### 2.5. Statistical Analysis:

Power was set at 80% and based on the ability to detect differences in group means equivalent to at least 1.2 standard deviations using a two-sample *t*-test. The Holm-Bonferroni method was used to control the family-wise error rate at 0.05 (for the tests shown in Table 1). Natural log transformations were used to normalize CXCL5, CXCL2, and CCL23 distributions. Where appropriate, nonparametric Mann-Whitney tests were substituted. Pearson’s correlation coefficient was used to identify associations between continuous measures. All p-values reported are two-sided. Statistical analyses were performed using RStudio^21^ and GraphPad Prism9 software.

## 3. Results

### 3.1. Screening analysis of serum cytokines-chemokines:

Using a 40-plex human chemokine assay kit, we measured the serum level of cytokines-chemokines from 11 healthy controls and 28 prostate cancer patients (14 each from CA and AA men). The descriptive analysis of serum concentration values for these cytokines-chemokines is provided in Supplementary Table 1. In this screening, when race-specific differences among cancer patients for a given cytokine or chemokine were not significantly different, we grouped such cases for an overall comparison between cancer patients and healthy controls. Independent of racial composition, serum levels of C-C chemokines CCL1 (*p* = *0.006*); CCL7 (*p* = *0.015*), CCL8 (*p* = *0.001*), CCL11 (*p* = *0.008*), CCL13 (*p* < *0.0001*), CXCL9 (*p* = *0.014*), CXCL10 (*p* = *0.043*), and the cytokine IL-2 (*p* = *0.047*) and TNF-α (*p* = *0.005*) were significantly lower in prostate cancer patients (n = 28) compared to healthy controls (n = 11). In contrast, race-specific differences were observed for significantly higher serum levels of CXCL2 (*p* < *0.0001*), CXCL5 (*p* = *0.016*), and IL-6 (*p* = *0.004*), and lower levels of CCL23 (*p* < *0.0001*) and CCL27 (*p* = *0.016*) in AA prostate cancer patients (n = 14) compared to CA (n = 14).

### 3.2. Serum chemokines profile and analysis summary:

**Figure 1.**
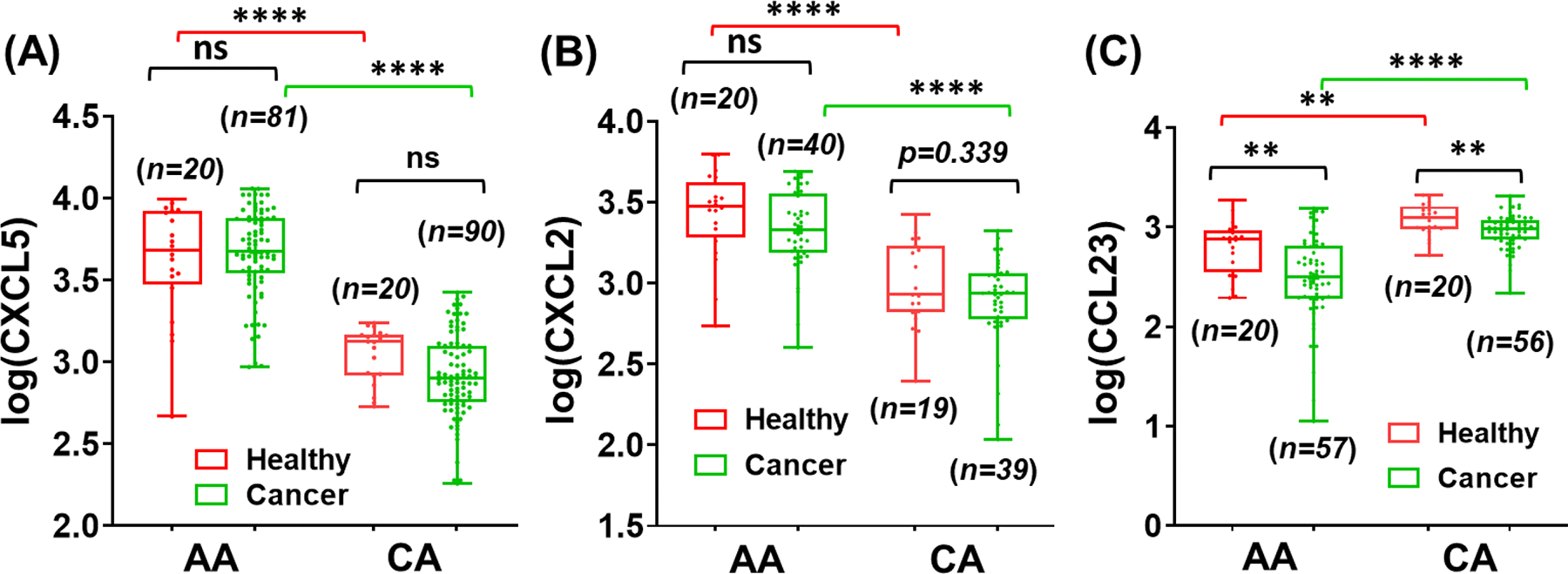
Race-associated differences in serum chemokines among AA and CA men. (A) CXCL5, (B) CXCL2, and (C) CCL23. Each dot represents a subject, and the error bars represent the mean ± SEM (standard error of the mean). Significance levels are *P < 0.05, **P < 0.01, ****P < 0.0001, ns = non significance. n = sample size per racial group.

Based on this screening, we validated that serum chemokines CXCL2, CXCL5, and CCL23 are the most distinguished chemokines differentially expressed in men of AA and CA races. The serum concentration values for these chemokines are presented in Table 1. Power for these analyses was based on the ability of a two-sample t-test to detect differences similar to those observed in the preliminary screening analyses. We used the Holm-Bonferroni method to control the family-wise error rate to 0.05 by adjusting the type-1 error rate for the 12 individual tests (Table 1). CXCL5 data analysis includes samples from 81 AA and 90 CA cases and 20 AA and 20 CA controls. The average age of all subjects was 58.5 years (SD = 8.8 years), and healthy controls were on average 7 years younger than cases (p < 0.001). Age accounted for less than 1% of the variation in log(CXCL5) (R^2^ = 0.005), and the association was the same for AA and CA. Among cases, AA males had Gleason scores 0.31 points higher (*p = 0.02*) and log(PSA) levels 0.23 points higher (*p = 0.04*) than CA males. Age, log(PSA), and Gleason scores were not significantly associated with log(CXCL5) values in cases (p > 0.5). Log(CXCL5) differed significantly by race, with AA having log(CXCL5) values 1.52 points higher than CA (*p < 0.001*). Log(CXCL5) did not differ between cases and controls once race differences were accounted for (*p = 0.4*). However, CA cases had slightly lower levels of CXCL5 than CA controls (Fig. 1A). A slight association of CXCL5 with disease status was found in CA, which did not appear to exist in AA. Likewise, the outcome observations for CXCL2 analysis corresponded to CXCL5 with only race-specific differences with significantly higher serum levels of CXCL2 in AA men than in CA (Fig. 1B).

CCL23 chemokine analysis consists of 77 and 76 samples each for AA and CA, including 20 healthy controls for each race. Multiple regression analysis revealed no association between the log(CCL23), age, log(PSA), and GS. Log(CCL23) differed significantly between the races with higher values in CA than AA samples (Fig. 1C). In AA cases, the CCL23 level was significantly lower (p < 0.01) than in healthy controls. CA cases also had slightly lower (2.96 ± 0.17) levels of CCL23 than CA controls (3.07 ± 0.16).

### 3.3. CXCL5 and CCL23 expression in archival specimens of human prostate tissues:

**Figure 2.**
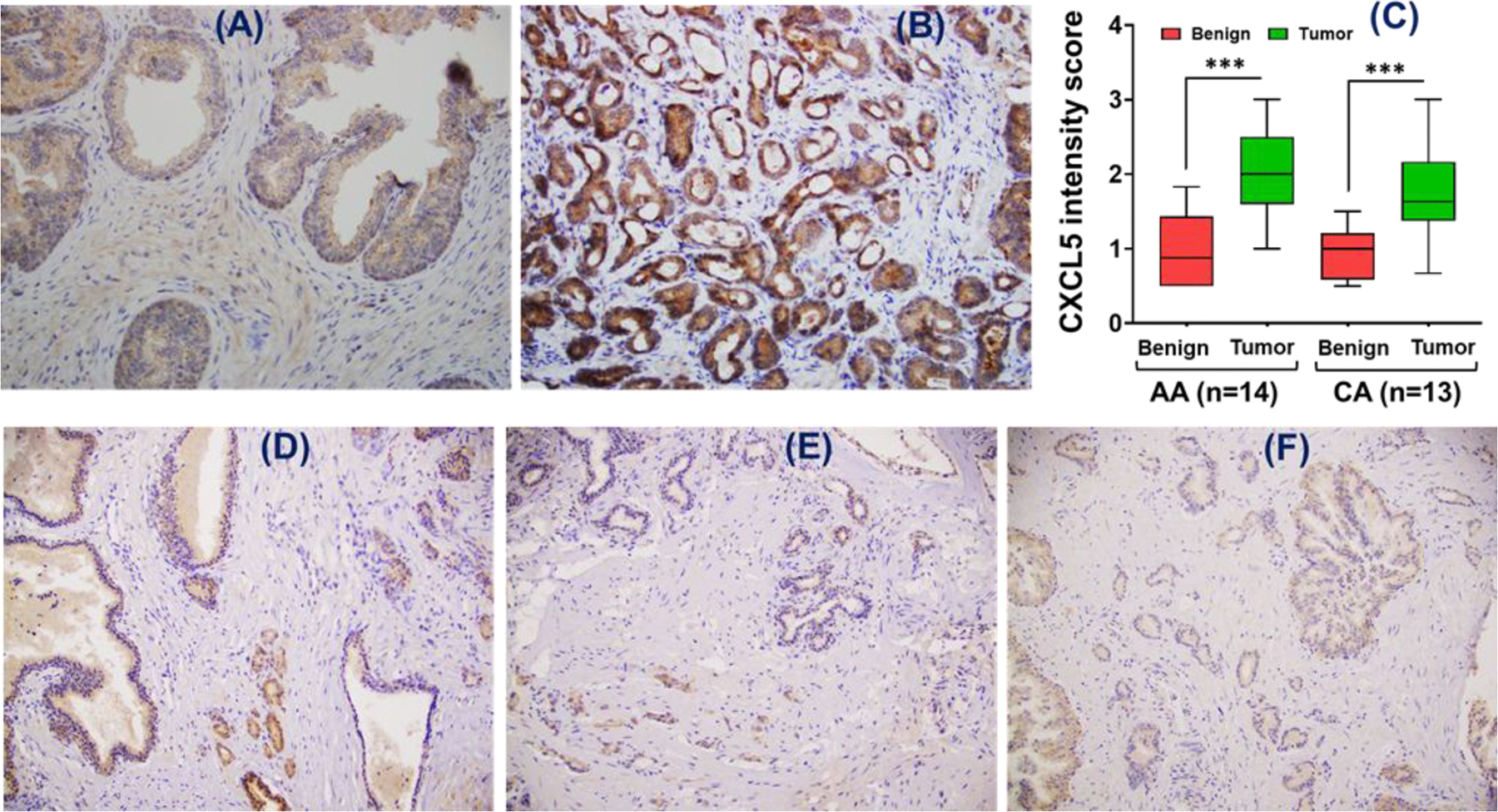
Representative monographs of CXCL5 (A-B) and CCL23 (D-F; AA: n = 10; CA: n = 10) expression in prostate tumor tissues. (A) benign; (B) cancer glands, (C) box and whisker plot for CXCL5 intensity score (AA: n = 14; CA: n = 13) with a significance level of ***P < 0.001, (D) cancer glands moderately positive than benign glands, (E) cancer glands positive and benign glands negative, and (F) cancer glands negative and benign glands with weak traces of cytoplasmic positivity.

To assess if CXCL5 and CCL23 are differentially expressed in prostate tumors, we performed immunohistochemistry (IHC) in archival prostate tumor tissues of AA and CA races. All cases of malignant tissues (AA: *n = 14* and CA: *n = 13*) on the tissue microarray (TMA) showed moderate to strong staining for CXCL5 expression, significantly higher (*p = <0.001*) than the adjacent benign tissues (Figs. 2A–2C). The mean value of CXCL5 intensity score in cancer was 2.03 ± 0.151 (mean ± SE) in AA compared to 1.77 ± 0.196 in CA cases, while the intensity of staining in the adjacent benign tissue for AA was 0.99 ± 0.134 compared to 0.92 ± 0.092 in CA cases. There was no indication of race-specific differences (*p = 0.62*) in this small set of cases. Similarly, no racial differences in CCL23 expression were observed in the analyzed 20 cases of prostate tumor tissues (10 each from AA and CA). Most cancer glands (13/20) were negative for CCL23 expression, while other cases showed a weak to moderate level of cytoplasmic positivity. All benign tissues showed weak traces of cytoplasmic CCL23 expression (Fig. 2D–2F).

## Discussion

In this study, we profiled the cytokine-chemokine serum levels associated with prostate cancer and demonstrated that serum chemokines CXCL2, CXCL5, and CCL23 are the most distinguished chemokines differentially expressed in men of AA and CA races.

Investigating the differential expression of race-specific cytokines among prostate cancer patients is of significant interest in potentially accounting for some of the variations in prostate cancer incidence and mortality between AA and CA men. Microarray-based gene expression analysis of larger sample sizes has shown increased expression of proinflammatory cytokines, including IFN-γ, TNF-α and interleukins (IL-1β, IL-6, and IL-8) in prostate tumor tissues of AA men than CAs^14,22^. However, our screening assay did not identify race-specific differences in serum TNF-α, IFN-γ, and IL-8 levels, whereas IL-6 level was significantly higher in prostate cancer men of AA race than CA. In healthy controls (n = 11), the median serum value of TNF-α (28.3 pg/ml) appeared to be higher than the normal range (8 – 10 pg/ml) in healthy men; however, the median values of IFN-γ (30.9 pg/ml) and IL-6 (7.3 pg/ml) seems within the normal range. Such differences could be attributed to the smaller sample size in our study or differences in the analytes and analysis techniques (serum ELISA vs. transcriptome profiling).

Chemokines are cell-secreted proteins with diverse physiological functions, including leukocyte trafficking, angiogenesis, inflammation, tumorigenesis, and metastasis. CXCL5, also known as ENA-78 (epithelial-derived neutrophil-activating peptide-78), is upregulated in many cancers^23–27^. Elevated CXCL5 protein expression in primary and metastatic prostate tumors aligned with prostate cancer promotion and metastasis, driving cell migration and EMT-transition in hormone-refractory prostate cancer cells^28,29^. A high circulatory level of CXCL5 impairs the functional activity of immune cells by suppressing the dendritic cell maturation signals and their functional activity^30^. In hepatocellular carcinoma, increased CXCL5 level in the tumor microenvironment (TME) was associated with PD-L1+ neutrophile infiltration, which diminished T cell function^31^. In addition, CXCL5 and CXCR2 ligand-receptor interaction drive infiltration of tumor-associated macrophages (TAMs) and myeloid-derived suppressor cells (MDSCs), promoting cancer metastasis in prostate cancer^32,33^.

As with CXCL5, our study also showed an increased serum CXCL2 level in AA men. A few studies signify the role of CXCL2 in promoting the progression and metastasis of cancer, including prostate, by inducing TAMs, recruitment of MDSCs, and suppression of CD8 T cell accumulation via CXCL2-CXCR2 signaling^34,35^. However, the functional role of CXCL2 in cancer between the races remained uncharacterized. Due to increased circulatory CXCL2 and CXCL5 levels, AA men likely acquire a disadvantage with vulnerable immune surveillance, leading to aggressive prostate cancer, as often seen in AA men at diagnosis.

CCL23, also known as macrophage inflammatory protein-3 (MIP-3), was the only observed chemokine showing race- and cancer-specific differences with significantly high serum levels of CCL23 in CA men than AA, while CCL23 was significantly low in cases with respective races. Previously, we hypothesized that CCL23 might be a stress-related chemokine and that an elevated CCL23 may help promote antitumor immunity^36^. Indeed, overexpression of CCL23 is associated with liver cancer suppression^37^, and an elevated serum level of CCL23 was linked with reduced risk factors in gastric cancer^38^. However, CCL23 is a relatively lesser characterized chemokine whose role in cancer suppression remains to be elucidated.

To our knowledge, there is no established normal range for serum concentration of CXCL2, CXCL5, and CCL23 in healthy males^39,40^. Such chemokines are primarily confined to laboratory research as an indicator for various diseases and are not routinely measured in clinical practice. For CXCL2, a median value of 301.44 (217.6 – 422.3) pg/ml is reported in healthy controls, whereas serum CXCL5 values ranged from 98.7 and up to ~3000 pg/ml^41–43^. Similarly, a recent study reported a CCL23 median value of 266.6 (32.1 – 652.5) pg/mL in healthy controls^44^. However, in these studies, the healthy controls include both males and females. It is worth pointing out that these values can vary widely among different laboratories with different testing methods. In our study, we analyzed the data using both parametric and nonparametric techniques, and the results remained consistent, demonstrating the robustness of our findings (Table 1). However, the clinical significance of these distinguished chemokines (CXLC2, CXCL5, and CCL23) needs further validation in a larger cohort of studies.

Keeping in view the established role of CXCL2 and CXCL5 in cancer progression and metastasis, our observations pose the question of whether the increased level of serum CXCL2 and CXCL5 in AA subjects (healthy controls and prostate cancer cases) is a consequence of genetic differences or results from environmental factors, including diet and lifestyle contribute to racial disparity in prostate cancer outcomes. A recent study of prostate cancer patients’ serum proteomic analysis noted a significantly high level of CXCL5 associated with West African ancestry^45^. It is reported that an increased circulatory CXCL5 in AA men could be accounted for by the lack of the duffy antigen receptor for chemokines (DARC), a receptor expressed on erythrocytes and vascular endothelial cells that functions as a biological sponge for the inflammatory chemokines, such as CXCL5^46–48^. As a genetic mechanism of protection against malarial infection, ~70% of the AA population lacks DARC expression in red blood cells^49–51^. Thus, the loss of DARC may be a potential trade-off in protecting against the malarial parasite with an increased level of inflammatory chemokine CXCL5 suppressing antitumor immunity and promoting aggressive prostate cancer in AA men. Like CXCL5, CXCL2 could be an ancestry-related chemokine; however, CCL23 is a novel chemokine that appeared to be independent of ancestry, and its role in tumor suppression warrants further investigation.

In summary, the elevated serum chemokines CXCL2 and CXCL5, in conjunction with a low level of CCL23, may contribute towards suppressed antitumor immunity and account for lethal prostate cancer in AA men. The observed disproportionate level of serum chemokines may help guide the therapeutic approaches in targeting aggressive prostate cancer when diagnosed in men of AA race. In addition, an antibody-directed neutralization of such chemokines might benefit prostate cancer treatment.

## Supplementary Material

Supplementary Table

## Figures and Tables

**Table 1.** Serum concentration values of chemokines in prostate cancer men of AA and Caucasian races compared to healthy controls.

Chemokine (pg/ml)	African American M ± SD (Range)		Caucasians M ± SD (Range)	
	Healthy Controls ^a^	Cancer ^b^	Healthy Controls ^c^	Cancer ^d^
**CXCL2**	3104.0 ± 1593.0 (545.5 – 6250.0)	2451.0 ± 1222.0 (400.0 – 4919.0)	1155.0 ± 658.0 (248.7 – 2642.0)	889.3 ± 433.9 (108.4 – 2114.0)
	*(n = 20)*	*(n = 40)*	*(n = 19)*	*(n = 39)*
*p-values*	^a^ *vs* ^b^ (*p = 0.108*); ^**a**^ ***vs*** ^**c**^ **(*p < 0.0001*)**; ^**b**^ ***vs*** ^**d**^ **(*p < 0.0001*)**; ^c^ *vs* ^d^ (*p = 0.339*)			
**CXCL5**	5189.0 ± 2939.0 (466.5 – 9818.0)	5459.0 ± 2785.0 (934.9 – 11474.0)	1183.0 ± 394.2 (535.6 – 1728)	977.5 ± 579.0 (181.6 – 2655.0)
	*(n = 20)*	*(n = 81)*	*(n = 20)*	*(n = 90)*
*p-values*	^a^ *vs* ^b^ (*p = 0.698*); ^**a**^ ***vs*** ^**c**^ **(*p < 0.0001*)**; ^**b**^ ***vs*** ^**d**^ **(*p < 0.0001*)**; ^**c**^ ***vs*** ^**d**^ **(*p = 0.023*)**			
**CCL23**	740.5 ± 423.7 (194.4 – 1860.0)	454.5 ± 392.5 (11.2 – 1554.0)	1263.0 ± 438.4 (523.5 – 2095.0)	966.6 ± 344.9 (217.9 – 2057.0)
	*(n = 20)*	*(n = 57)*	*(n = 20)*	*(n = 56)*
*p-values*	^**a**^ ***vs*** ^**b**^ **(*p = 0.002*);** ^**a**^ ***vs*** ^**c**^ **(*p = 0.0004*);** ^**b**^ ***vs*** ^**d**^ **(*p < 0.0001*);** ^**c**^ ***vs*** ^**d**^ **(*p = 0.009*)**			

*p-values* are based on nonparametric Mann-Whitney test. M ± SD: standard deviation of the mean; *n* is the number of subjects.

## Data Availability

All data to support the proposed study is included in this publication.

## References

[1] Siegel RL, Miller KD, Fuchs HE, Jemal A. Cancer statistics, 2022. CA Cancer J Clin. 2022;72(1):7–33. 35020204 10.3322/caac.21708

[2] Giaquinto AN, Miller KD, Tossas KY, Winn RA, Jemal A, Siegel RL. Cancer statistics for African American/Black People 2022. CA Cancer J Clin. 2022. 10.3322/caac.21718 35143040

[3] Dess RT, Hartman HE, Mahal BA, Association of Black Race With Prostate Cancer-Specific and Other-Cause Mortality. JAMA Oncol. 2019;5(7):975–983. 31120534 10.1001/jamaoncol.2019.0826 PMC6547116

[4] Paller CJ, Wang L, Brawley OW. Racial Inequality in Prostate Cancer Outcomes-Socioeconomics, Not Biology. JAMA Oncol. 2019;5(7):983–984. 31120499 10.1001/jamaoncol.2019.0812 PMC9195566

[5] Stern N, Ly TL, Welk B, Association of Race and Ethnicity With Prostate Cancer-Specific Mortality in Canada. JAMA Netw Open. 2021;4(12):e2136364. 34932109 10.1001/jamanetworkopen.2021.36364 PMC8693210

[6] Johnson JR, Woods-Burnham L, Hooker SE Jr., Batai K, Kittles RA. Genetic Contributions to Prostate Cancer Disparities in Men of West African Descent. Front Oncol. 2021;11:770500. 34820334 10.3389/fonc.2021.770500 PMC8606679

[7] Smith ZL, Eggener SE, Murphy AB. African-American Prostate Cancer Disparities. Curr Urol Rep. 2017;18(10):81. 28808871 10.1007/s11934-017-0724-5

[8] Stabellini N, Cullen J, Bittencourt MS, Allostatic load and cardiovascular outcomes in males with prostate cancer. JNCI Cancer Spectr. 2023. 10.1093/jncics/pkad005 PMC10005613 36752520

[9] Hughes Halbert C, Jefferson M, Ambrose L, Caulder S, Savage SJ. Resiliency and Allostatic Load among Veterans at Risk for Adverse Prostate Cancer Outcomes. Ethn Dis. 2020;30(Suppl 1):177–184. 32269459 10.18865/ed.30.S1.177 PMC7138440

[10] Kiely M, Lord B, Ambs S. Immune response and inflammation in cancer health disparities. Trends Cancer. 2021. 10.1016/j.trecan.2021.11.010 PMC8930618 34965905

[11] Yao S, Cheng TD, Elkhanany A, Breast Tumor Microenvironment in Black Women: A Distinct Signature of CD8+ T-Cell Exhaustion. J Natl Cancer Inst. 2021;113(8):1036–1043. 33395700 10.1093/jnci/djaa215 PMC8328978

[12] Meaney CL, Mitchell KA, Zingone A, Circulating Inflammation Proteins Associated With Lung Cancer in African Americans. J Thorac Oncol. 2019;14(7):1192–1203. 30953795 10.1016/j.jtho.2019.03.014 PMC6592767

[13] Wallace TA, Prueitt RL, Yi M, Tumor immunobiological differences in prostate cancer between African-American and European-American men. Cancer Res. 2008;68(3):927–936. 18245496 10.1158/0008-5472.CAN-07-2608

[14] Awasthi S, Berglund A, Abraham-Miranda J, Comparative Genomics Reveals Distinct Immune-oncologic Pathways in African American Men with Prostate Cancer. Clin Cancer Res. 2021;27(1):320–329. 33037017 10.1158/1078-0432.CCR-20-2925 PMC8042600

[15] Karan D, Wick J, Dubey S, Tawfik O, Van Veldhuizen P. Circulatory MIC-1 as a Determinant of Prostate Cancer Racial Disparity. Cancers (Basel). 2020;12(10). 10.3390/cancers12103033 PMC7603134 33081054

[16] Kiely M, Ambs S. Immune Inflammation Pathways as Therapeutic Targets to Reduce Lethal Prostate Cancer in African American Men. Cancers (Basel). 2021;13(12). 10.3390/cancers13122874 PMC8227648 34207505

[17] Hoffmann SC, Stanley EM, Cox ED, Ethnicity greatly influences cytokine gene polymorphism distribution. Am J Transplant. 2002;2(6):560–567. 12118901 10.1034/j.1600-6143.2002.20611.x

[18] Longo DM, Louie B, Mathi K, Racial differences in B cell receptor signaling pathway activation. J Transl Med. 2012;10:113. 22672557 10.1186/1479-5876-10-113 PMC3464787

[19] Gillard M, Javier R, Ji Y, Elevation of Stromal-Derived Mediators of Inflammation Promote Prostate Cancer Progression in African-American Men. Cancer Res. 2018;78(21):6134–6145. 30181178 10.1158/0008-5472.CAN-17-3810

[20] Hawley JE, Pan S, Kandadi H, Chaimowitz MG, Sheikh N, Drake CG. Analysis of Circulating Immune Biomarkers by Race in Men With Metastatic Castration-Resistant Prostate Cancer Treated With Sipuleucel-T. J Natl Cancer Inst. 2022;114(2):314–317. 34302463 10.1093/jnci/djab145 PMC8826456

[21] RStudio T RStudio: Integrated Development for R. RStdio, PBC, Boston, MA. 2021.

[22] Powell IJ, Dyson G, Land S, Genes associated with prostate cancer are differentially expressed in African American and European American men. Cancer Epidemiol Biomarkers Prev. 2013;22(5):891–897. 23515145 10.1158/1055-9965.EPI-12-1238 PMC4097306

[23] Hsu YL, Hou MF, Kuo PL, Huang YF, Tsai EM. Breast tumor-associated osteoblast-derived CXCL5 increases cancer progression by ERK/MSK1/Elk-1/snail signaling pathway. Oncogene. 2013;32(37):4436–4447. 23045282 10.1038/onc.2012.444

[24] Zhou X, Peng M, He Y, CXC Chemokines as Therapeutic Targets and Prognostic Biomarkers in Skin Cutaneous Melanoma Microenvironment. Front Oncol. 2021;11:619003. 33767987 10.3389/fonc.2021.619003 PMC7985846

[25] Li A, King J, Moro A, Overexpression of CXCL5 is associated with poor survival in patients with pancreatic cancer. Am J Pathol. 2011;178(3):1340–1349. 21356384 10.1016/j.ajpath.2010.11.058 PMC3069811

[26] Kawamura M, Toiyama Y, Tanaka K, CXCL5, a promoter of cell proliferation, migration and invasion, is a novel serum prognostic marker in patients with colorectal cancer. Eur J Cancer. 2012;48(14):2244–2251. 22197219 10.1016/j.ejca.2011.11.032

[27] Zheng J, Zhu X, Zhang J. CXCL5 knockdown expression inhibits human bladder cancer T24 cells proliferation and migration. Biochem Biophys Res Commun. 2014;446(1):18–24. 24583128 10.1016/j.bbrc.2014.01.172

[28] Begley LA, Kasina S, Mehra R, CXCL5 promotes prostate cancer progression. Neoplasia. 2008;10(3):244–254. 18320069 10.1593/neo.07976 PMC2262133

[29] Kuo PL, Chen YH, Chen TC, Shen KH, Hsu YL. CXCL5/ENA78 increased cell migration and epithelial-to-mesenchymal transition of hormone-independent prostate cancer by early growth response-1/snail signaling pathway. J Cell Physiol. 2011;226(5):1224–1231. 20945384 10.1002/jcp.22445

[30] Michielsen AJ, Hogan AE, Marry J, Tumour tissue microenvironment can inhibit dendritic cell maturation in colorectal cancer. PLoS One. 2011;6(11):e27944. 22125641 10.1371/journal.pone.0027944 PMC3220715

[31] Deng H, Kan A, Lyu N, Tumor-derived lactate inhibit the efficacy of lenvatinib through regulating PD-L1 expression on neutrophil in hepatocellular carcinoma. J Immunother Cancer. 2021;9(6). 10.1136/jitc-2020-002305 PMC8231064 34168004

[32] Wang G, Lu X, Dey P, Targeting YAP-Dependent MDSC Infiltration Impairs Tumor Progression. Cancer Discov. 2016;6(1):80–95. 26701088 10.1158/2159-8290.CD-15-0224 PMC4707102

[33] Li BH, Garstka MA, Li ZF. Chemokines and their receptors promoting the recruitment of myeloid-derived suppressor cells into the tumor. Mol Immunol. 2020;117:201–215. 31835202 10.1016/j.molimm.2019.11.014

[34] Di Mitri D, Mirenda M, Vasilevska J, Re-education of Tumor-Associated Macrophages by CXCR2 Blockade Drives Senescence and Tumor Inhibition in Advanced Prostate Cancer. Cell Rep. 2019;28(8):2156–2168 e2155. 31433989 10.1016/j.celrep.2019.07.068 PMC6715643

[35] Hu J, Zhao Q, Kong LY, Regulation of tumor immune suppression and cancer cell survival by CXCL1/2 elevation in glioblastoma multiforme. Sci Adv. 2021;7(5). 10.1126/sciadv.abc2511 PMC7840139 33571109

[36] Karan D CCL23 in Balancing the Act of Endoplasmic Reticulum Stress and Antitumor Immunity in Hepatocellular Carcinoma. Front Oncol. 2021;11:727583. 34671553 10.3389/fonc.2021.727583 PMC8522494

[37] Meng J, Wang L, Hou J, CCL23 suppresses liver cancer progression through the CCR1/AKT/ESR1 feedback loop. Cancer Sci. 2021;112(8):3099–3110. 34050704 10.1111/cas.14995 PMC8353945

[38] Camargo MC, Song M, Sawada N, Prediagnostic circulating inflammation-related biomarkers and gastric cancer: A case-cohort study in Japan. Cytokine. 2021;144:155558. 33985855 10.1016/j.cyto.2021.155558 PMC13539773

[39] Costantini S, Capone F, Miele M, CytokineDB: a database collecting biological information. Bioinformation. 2009;4(3):92–93. 20198179 10.6026/97320630004092 PMC2828895

[40] Miele M, Sharma A, Capone F, CytReD: A database collecting human cytokinome information. Bioinformation. 2011;6(5):207–208. 21738316 10.6026/97320630006207 PMC3124794

[41] Singh UP, Singh NP, Murphy EA, Chemokine and cytokine levels in inflammatory bowel disease patients. Cytokine. 2016;77:44–49. 26520877 10.1016/j.cyto.2015.10.008 PMC4666758

[42] Tacke F, Zimmermann HW, Trautwein C, Schnabl B. CXCL5 plasma levels decrease in patients with chronic liver disease. J Gastroenterol Hepatol. 2011;26(3):523–529. 21332547 10.1111/j.1440-1746.2010.06436.x PMC3058722

[43] Wang X, Sun L, He N, Increased expression of CXCL2 in ACPA-positive rheumatoid arthritis and its role in osteoclastogenesis. Clin Exp Immunol. 2021;203(2):194–208. 33010041 10.1111/cei.13527 PMC7806446

[44] Roderburg C, Labuhn S, Bednarsch J, Elevated Serum Levels of CCL23 Are Associated with Poor Outcome after Resection of Biliary Tract Cancer. Mediators Inflamm. 2022;2022:6195004. 36505756 10.1155/2022/6195004 PMC9731746

[45] Minas TZ, Candia J, Dorsey TH, Serum proteomics links suppression of tumor immunity to ancestry and lethal prostate cancer. Nat Commun. 2022;13(1):1759. 35365620 10.1038/s41467-022-29235-2 PMC8975871

[46] Dawson TC, Lentsch AB, Wang Z, Exaggerated response to endotoxin in mice lacking the Duffy antigen/receptor for chemokines (DARC). Blood. 2000;96(5):1681–1684. 10961863

[47] Novitzky-Basso I, Rot A. Duffy antigen receptor for chemokines and its involvement in patterning and control of inflammatory chemokines. Front Immunol. 2012;3:266. 22912641 10.3389/fimmu.2012.00266 PMC3421148

[48] Gardner L, Patterson AM, Ashton BA, Stone MA, Middleton J. The human Duffy antigen binds selected inflammatory but not homeostatic chemokines. Biochem Biophys Res Commun. 2004;321(2):306–312. 15358176 10.1016/j.bbrc.2004.06.146

[49] Lentsch AB. The Duffy antigen/receptor for chemokines (DARC) and prostate cancer. A role as clear as black and white? FASEB J. 2002;16(9):1093–1095. 12087071 10.1096/fj.02-0066hyp

[50] Miller LH, Mason SJ, Clyde DF, McGinniss MH. The resistance factor to Plasmodium vivax in blacks. The Duffy-blood-group genotype, FyFy. N Engl J Med. 1976;295(6):302–304. 778616 10.1056/NEJM197608052950602

[51] Shen H, Schuster R, Stringer KF, Waltz SE, Lentsch AB. The Duffy antigen/receptor for chemokines (DARC) regulates prostate tumor growth. FASEB J. 2006;20(1):59–64. 16394268 10.1096/fj.05-4764com

