# Supplementary material for: Racial differences in serum chemokines in prostate cancer patients
Source: Cancer. Author manuscript; Available in PMC 2025 Sep 9. (PMC12415822; doi:10.1002/cncr.35012)
Supplement: Supplementary Table [file NIHMS2106488-supplement-Supplementary_Table.docx]

**Supplementary Table 1.** Serum concentration values of chemokines and cytokines in healthy controls and prostate cancer men of African American (AA) and Caucasian (CA) races based on the 40-plex assay.

| Chemokine/ Cytokine | CA: Healthy Controls (n=11)  M ± SD (Range) | CA: Cancer (n = 14)  M ± SD (Range) | *CA-Control vs. CA-Cancer*  *(p-value)* | AA: Cancer (n = 14)  M ± SD (Range) | AA-Cancer *vs.* CA-Cancer  *(p-value)* |
| --- | --- | --- | --- | --- | --- |
| **CCL1**  **CCL2**  **CCL3**  **CCL7**  **CCL8**  **CCL11**  **CCL13**  **CCL15**  **CCL17**  **CCL19**  **CCL20**  **CCL21**  **CCL22**  **CCL23**  **CCL24**  **CCL25**  **CCL26**  **CCL27**  **CX3CL1**  **CXCL1**  **CXCL2**  **CXCL5**  **CXCL6**  **CXCL9**  **CXCL10**  **CXCL11**  **CXCL12**  **CXCL13**  **CXCL16**  **IL-1b**  **IL-2**  **IL-4**  **IL-6**  **IL-8**  **IL-10**  **IL-16**  **IFN-γ**  **TNF-α**  **GM-CSF**  **MIF** | 47.0 ± 7.3 (36.8 - 58.4)  79.9 ± 47.9 (14.1 - 147.7)  8.7 ± 2.5 (5.8 - 13.3)  69.1 ± 13.7 (48.1 - 92.5)  82.7 ± 24.0 (19.8 - 106.5)  44.4 ± 11.4 (32.0 - 63.8)  145.4 ± 87.2 (89.9 - 398.0)  19665 ± 20959 (1541 - 54315)  262.6 ± 209.6 (81.1 - 731.1)  671.8 ± 387.4 (292.6 - 1415.0)  8.3 ± 2.2 (5.9 - 12.4)  4594 ± 2164 (1751 - 9115)  900.4 ± 243.4 (641.2 - 1441.0)  208.4 ± 169.6 (17.4 - 514.6)  391.0 ± 248.8 (42.1 - 968.2)  754.4 ± 218.7 (442.1 - 1082.0)  44.2 ± 11.4 (31.5 - 65.9)  1422 ± 662.1 (383.4 - 2424.0)  237.3 ± 107.5 (136.3 - 460.8)  254.2 ± 132.3 (164.1 - 622.2)  468.6 ± 438.6 (36.0 - 1540.0)  600.5 ± 656.3 (7.2 - 2370.0)  34.9 ± 12.5 (13.0 - 51.2)  539.9 ± 415.3 (292.1 - 1709.0)  223.3 ± 169.8 (50.8 - 599.4)  50.5 ± 38.8 (9.9 - 134.0)  1306 ± 608.4 (118.0 - 2194.0)  19.3 ± 7.1 (10.3 - 31.2)  483.9 ± 138.5 (192.1 - 658.4)  3.1 ± 1.3 (2.2 - 6.8)  9.5 ± 2.7 (5.9 - 14.5)  11.6 ± 2.9 (6.7 - 16.7)  7.8 ± 2.1 (4.8 - 10.9)  17.8 ± 21.4 (6.5 - 81.6)  39.6 ± 11.5 (27.1 - 60.2)  307.2 ± 72.5 (233.0 - 461.9)  35.0 ± 9.1 (24.3 - 51.9)  28.4 ± 4.3 (19.6 - 34.8)  26.5 ± 11.9 (9.3 - 49.5)  11083 ± 9979 (1869 - 27235) | 39.9 ± 11.8 (23.6 - 63.1)  76.3 ± 36.1 (2.9 - 143.0)  6.8 ± 2.9 (3.2 - 14.3)  55.4 ± 22.0 (24.2 - 94.3)  43.6 ± 15.4 (12.2 - 70.6)  37.9 ± 19.4 (13.9 - 79.6)  74.9 ± 45.9 (12.3 - 173.8)  7400 ± 3993 (2619 - 16539)  213.8 ± 228.5 (16.7 - 831.8)  613.9 ± 604.5 (52.7 - 2004.0)  6.96 ± 3.26 (2.5 - 12.9)  5944 ± 2922 (1377 - 10871)  813.6 ± 542.0 (97.5 - 2342)  **332.1 ± 149.1 (69.9 - 504.7)**  545.5 ± 379.1 (12.2 - 1256.0)  833.5 ± 718.8 (274.7 - 3153.0)  43.2 ± 19.9 (15.7 - 83.9)  **1191.0 ± 515.8 (302.0 - 1983.0)**  184.1 ± 69.0 (60.5 - 362.7)  167.8 ± 56.3 (81.9 - 277.2)  **260.4 ± 231.0 (25.6 - 782.3)**  **445.0 ± 219.4 (187.3 - 948.7)**  29.6 ± 12.6 (14.2 - 59.0)  413.6 ± 297.2 (112.6 - 1108.0)  148.3 ± 81.5 (61.9 - 340.2)  30.1 ± 17.9 (12.3 - 77.5)  1225.0 ± 697 (113.8 - 2477.0)  21.1 ± 9.8 (7.2 - 34.4)  459.7 ± 127.2 (196.3 - 672.9)  2.5 ± 1.0 (1.1 - 4.2)  8.2 ± 4.9 (2.5 - 19.6)  10.8 ± 5.0 (2.6 - 17.9)  **13.6 ± 9.7 (3.4 - 35.8)**  14.4 ± 18.9 (3.2 - 78.6)  32.7 ± 18.6 (8.9 - 60.9)  491.4 ± 232.2 (117.2 - 906.1)  29.7 ± 12.0 (13.7 - 50.6)  23.5 ± 7.9 (13.0 - 36.9)  33.5 ± 15.9 (17.2 - 65.4)  7576.0 ± 6863.0 (1573 - 21788) | 0.134  0.999  0.095  0.170  ***0.0002***  0.130  ***0.004***  0.625  0.344  0.536  0.312  0.202  0.267  0.075  0.373  0.609  0.896  0.403  0.244  ***0.009***  0.166  0.797  0.244  0.267  0.244  0.223  0.687  0.686  0.686  0.217  0.267  0.936  ***0.044***  0.250  0.317  ***0.051***  0.292  0.075  0.434  0.483 | 36.0 ± 7.8 (17.9 - 49.7)  80.9 ± 72.4 (26.0 - 303.1)  35.9 ± 77.8 (4.0 - 297.8)  51.3 ± 14.7 (17.4 - 86.1)  68.8 ± 43.0 (11.9 - 189.9)  27.3 ± 10.2 (14.1 - 50.6)  60.9 ± 49.3 (24.9 - 217.2)  17992 ± 16074 (3122 - 45516)  210.2 ± 308.3 (46.6 - 1269.0)  491.0 ± 515.1 (53.0 - 2196.0)  5.9 ± 3.1 (2.6 - 14.3)  5214 ± 2642 (1915 - 10708)  668.6 ± 280.1 (293.7 - 1336)  **104.6 ± 119.3 (8.4 - 382.2)**  278.8 ± 165.6 (34.6 - 664.0)  571.5 ± 234.4 (313.1 - 1287.0)  33.1 ± 12.9 (17.2 - 61.3)  **779.8 ± 267.3 (363.4 - 1454.0)**  174.0 ± 68.3 (115.4 - 365.3)  276.0 ± 194.9 (66.5 - 840.5)  **1073.0 ± 818.4 (79.6 - 2679.0)**  **1154.0 ± 906.2 (266.9 - 3038.0)**  45.1 ± 30.4 (5.9 - 101.6)  288.9 ± 152.2 (101.7 - 755.7)  106.6 ± 69.1 (20.4 - 276.3)  33.9 ± 30.9 (5.7 - 108.8)  1084.0 ± 232.5 (678.2 - 1480.0)  17.3 ± 7.8 (6.6 - 37.6)  483.9 ± 88.5 (386.7 - 703.1)  2.3 ± 0.7 (0.9 - 3.6)  6.9 ± 2.4 (1.7 - 12.7)  8.4 ± 2.9 (2.4 - 13.7)  **28.9 ± 19.5 (9.1 - 83.7)**  22.1 ± 37.5 (3.9 - 150.1)  33.3 ± 14.9 (7.9 - 66.4)  455.0 ± 382.4 (131.8 - 1334)  27.7 ± 8.1 (11.9 - 48.6)  21.4 ± 5.9 (9.5 - 36.2)  32.7 ± 10.9 (9.3 - 56.2)  4675.0 ± 5552.0 (1153 - 22615) | 0.312  0.401  0.140  0.563  0.085  0.111  0.328  0.106  0.874  0.946  0.382  0.427  0.376  ***<0.0001***  0.085  0.210  0.265  ***0.016***  0.401  0.094  ***<0.0001***  ***0.016***  0.194  0.227  0.094  0.769  0.999  0.376  0.667  0.667  0.635  0.174  ***0.004***  0.482  0.804  0.306  0.603  0.454  0.583  0.085 |

*p-values* are based on nonparametric Mann-Whitney test. M ± SD: standard deviation of the mean; *n* is the number of subjects.
